# Is motor inhibition involved in the processing of sentential negation? An assessment via the Stop-Signal Task

**DOI:** 10.1007/s00426-021-01512-7

**Published:** 2021-04-27

**Authors:** Martina Montalti, Marta Calbi, Valentina Cuccio, Maria Alessandra Umiltà, Vittorio Gallese

**Affiliations:** 1 Department of Medicine and Surgery Unit of Neuroscience, University of Parma, Parma, Italy; 2 Department of Cognitive, Psychological, Pedagogical Sciences and Cultural Studies, University of Messina, Messina, Italy; 3 Department of Food and Drug, University of Parma, Parma, Italy; 4 Berlin School of Mind and Brain, Humboldt-Universität Zu Berlin, Berlin, Germany

## Abstract

In the last decades, the embodied approach to cognition and language gained momentum in the scientific debate, leading to evidence in different aspects of language processing. However, while the bodily grounding of concrete concepts seems to be relatively not controversial, abstract aspects, like the negation logical operator, are still today one of the main challenges for this research paradigm. In this framework, the present study has a twofold aim: (1) to assess whether mechanisms for motor inhibition underpin the processing of sentential negation, thus, providing evidence for a bodily grounding of this logic operator, (2) to determine whether the Stop-Signal Task, which has been used to investigate motor inhibition, could represent a good tool to explore this issue. Twenty-three participants were recruited in this experiment. Ten hand-action-related sentences, both in affirmative and negative polarity, were presented on a screen. Participants were instructed to respond as quickly and accurately as possible to the direction of the Go Stimulus (an arrow) and to withhold their response when they heard a sound following the arrow. This paradigm allows estimating the Stop Signal Reaction Time (SSRT), a covert reaction time underlying the inhibitory process. Our results show that the SSRT measured after reading negative sentences are longer than after reading affirmative ones, highlighting the recruitment of inhibitory mechanisms while processing negative sentences. Furthermore, our methodological considerations suggest that the Stop-Signal Task is a good paradigm to assess motor inhibition’s role in the processing of sentence negation.

**Supplementary Information:**

The online version contains supplementary material available at 10.1007/s00426-021-01512-7.

## Introduction

The embodied account of language, which claims the involvement of the sensory-motor system in linguistic processing (for a review, see Cuccio & Gallese, 2018; Gallese, 2008), is today a prominent approach to the explanation of language understanding that refuses the classical cognitive science view based on the idea that concepts and meanings are represented using amodal and abstract symbols (e.g., Fodor, 1983; Pylyshyn, 1984). To date, a huge amount of experimental studies, carried out with several experimental techniques, have supported this embodied approach (for a review, see Jirak et al., 2010) and have shown that the sensory-motor system is recruited by different aspects of language processing such as phonological processing (D'Ausilio et al., 2009; Di Cesare et al., 2017; Hickok, 2010; Pulvermüller, 2018), semantic processing—for both action-related (Buccino et al., 2005; Gallese & Cuccio, 2018; Mirabella et al., 2012b; Sato et al., 2008; Spadacenta et al., 2014; Tettamanti et al., 2005) and abstract-related words/sentences (Borghi et al., 2017; Cuccio & Gallese, 2018; Cuccio & Caruana, 2019; Dreyer & Pulvermüller, 2018) and even pragmatic aspects of communication (Cuccio et al., 2014; Egorova et al., 2016). Moreover, it is known that in the action language interaction, motor simulation can lead both to facilitation or interference effects depending on the extent of the temporal overlapping between linguistic and motor tasks (Shebani & Pulvermüller, 2018). Processing action-related language interferes with the execution of a concurrent motor act; whereas, it facilitates the performance of a subsequent movement if it is performed prior to the movement onset. A facilitation effect will determine shorter reaction times (RTs) in the performance of the motor act; whereas, the interference effect will lead to longer RTs. Furthermore, facilitation and interference effects can also be modulated by factors such as sentence-structures features (Borghi et al., 2010; Borghi, 2012; Candidi et al., 2010) like verb tense and the pronoun. Indeed, it is demonstrated that the imperative tense is the strongest form for generating a motor preparation process since it leads to take on an agent role. As for the perspective, there is evidence that the use of the second person leads to assuming the agent point of view and, thus, this determines a facilitation effect; whereas, an interference effect is frequently demonstrated using the first and the third person (for a review Borghi et al., 2017).

From a developmental perspective, the embodied approach to language suggests that the acquisition of word meanings develops from internal simulations of the experiences linked to the word, which are reflected in the reactivation of the same neural areas involved when we experience the concepts expressed by the words (Barsalou, 1999; Gallese, 2008; Glenberg & Gallese, 2012).

Whereas, the functioning of this simulation mechanism may be relatively clear and straightforward with regard to the processing of concrete concepts and words, when it comes to abstract words and concepts (i.e., concepts that do not designate referents that can be perceived through our body), it becomes highly problematic. Indeed, although there is evidence of an involvement of the motor system during the processing of abstract words (e.g., Cuccio & Caruana, 2019; Grade et al., 2017; Mazzuca et al., 2018; for a review see Borghi & Zarcone, 2016; Borghi et al., 2017), the debate is still open and highly controversial results have been reported (for a review, see Borghi et al., 2017).

There are different ways in which this topic can be tackled. On the one hand, abstract words or sentences with metaphorical meaning can be exploited instead of using concrete action-related-words or sentences with concrete action-related-meaning (for a review, see Cuccio, 2018). On the other hand, the processing of abstract linguistic structures such as logic operators can be a pivotal test to assess whether the embodied theory of language can be extended to abstract language. One prominent example of an abstract linguistic structure is the sentential negation.

Negation allows inverting the truth-value of a sentence (Cuccio, 2011, 2012; Horn, 1989). This represents a characteristic and fundamental feature of human communication, letting us perform mathematical reasoning and philosophical hypothesis, create counterfactual reasoning, and discuss ethics. With regard to the bodily grounding of negation, it could be argued that the same mechanisms subserving motor inhibition (Mirabella, 2014) are involved in the understanding of the negation of action-related sentences. If this is the case, then a motor routine would turn out to be exploited for language comprehension, pointing out a tight coupling between motor and linguistic processes (Gallese, 2008).

In the last decades, several studies investigated the cognitive effects of sentence negation, demonstrating its association with both an increased cognitive effort and a reduced accessibility of the negated concepts during their processing with respect to the affirmative counterpart. These phenomena are reflected in higher error rates and longer RTs (Clark & Chase, 1972; Carpenter & Just, 1975; Kaup, 2001; Kaup & Zwaan, 2003; MacDonald & Just, 1989). Furthermore, it has also been shown that negation elicits complementary scenarios (Kaup et al., 2005; Orenes et al., 2014). In the last few years, several studies aimed to understand the neural underpinnings of sentence negation processing. All in all, they provide evidence for a modulation of the motor and pre-motor cortex according to the polarity of the sentence, i.e., they showed reduced activation of the hand-motor areas during the processing of hand-action-related negative sentences, with respect to their affirmative counterpart (Alemanno et al., 2012; Aravena et al., 2012; Bartoli et al., 2013; Foroni & Semin, 2013; Liuzza et al., 2011; Tettamanti et al., 2008; Tomasino et al., 2010). These results seem to support the embodied account of language, but the neurocognitive mechanisms underlying the processing of sentential negation have not been understood yet.

Recently, it has been proposed that a good candidate for this role is the neural mechanism of motor response inhibition (Beltrán et al., 2018; Beltrán et al., 2019; Foroni & Semin, 2013; García-Marco et al., 2019; Liu et al., 2019; Papeo et al., 2016; de Vega et al., 2016). Inhibitory control represents a crucial executive function, which allows the implementation of adaptive and flexible behavioural strategies (Chambers et al., 2009; Mirabella, 2014; Matzke et al., 2018).

Response inhibition is a multifaceted executive function: in the present paper, we will focus on motor inhibition, i.e., the ability to inhibit a prepotent motor response, because it represents the most likely mechanism involved in the processing of action-related sentence negation. As motor inhibition is frequently assessed via the Stop Signal task (SST, Logan et al., 1984), we will exploit this paradigm to test the embodied theory of language.

### The Stop-Signal paradigm

For the sake of clarity and to clarify the reasons that led us to develop the present project, in this section we will present the main features of the experimental paradigm and, as far as we know, the only other study that used it to investigate sentence negation (Beltrán et al., 2018).

The SST probes the participants’ ability to cancel a pre-planned movement when an infrequent stop instruction is presented at some delay after the presentation of a go-signal. Thus, the SST consists of two tasks. A Go- (a RT-task), and a Stop-task. Trials of the two tasks are randomly intermixed. The Go trials are the most frequent type of trials and require the subject to respond as fast as possible when a Go-signal is presented. The Stop-Signal trials are less frequent and require the subject to cancel the response triggered by the Go-signal at the presentation of a Stop-Signal. The SST allows computing reactive inhibition, i.e., the ability of subjects to react to a stop-signal, via the SSRT, which is estimated using the race model (Logan et al., 1984). Some versions of the SST also allow to estimate proactive inhibition, i.e. the ability of participants to shape their response strategies according to the current context. Proactive inhibition can be assessed either by comparing the RTs (Chikazoe et al., 2009; Majid et al., 2013; Zandbelt & Vink, 2010 ) or both the RTs and the movement times (Mancini et al., 2018; Mirabella et al., 2008) between two or more conditions, which require different degrees of alertness for the presentation of the Stop-Signal.

The frequency of Stop-Signal trials is crucial because as it increases, the probability of using waiting strategies increases, i.e., slowing down the movement and delaying the response to increase the probability of suppressing it when a stop-signal is presented (Federico & Mirabella, 2014; Logan, 1981; Logan & Burkell, 1986; Ramautar et al., 2004). Generally, the Stop-Signal probability should range between 0.1 and 0.3 (Matzke et al., 2018), considering 0.25 a good compromise (Logan, 1994; Verbruggen et al., 2019).

According to the independent horse-race model (Logan et al., 1984; Matzke et al., 2018; Verbruggen et al., 2019), under the Go- and the Stop-task there are two independent processes: a Go process and a Stop process, triggered by the presentation of the Go stimulus and the Stop-Signal, respectively. Like a race, the process that ends first wins, leading to the success (i.e., Stop process wins and no response is emitted) or to the failure (i.e., Go process wins and a response is incorrectly emitted) of the inhibition task. The first assumption of the model is the Independence Assumption, which states that: (i) Go- and Stop processes are independent one of another (Stochastic Independence); and (ii) the RTs distributions of the Go process in Go trials and Stop trials are the same (Context Independence). The second assumption is that SSRT is assumed to be constant. The SSRT estimation is based on three variables: the Go RTs distribution, the probability to respond to the signal, and the Stop Signal Delay (SSD). Indeed, the stop-signal’s onset is dynamically adjusted according to the performance of the participant. When (s)he is able to properly suppress the imminent response to the Go trial, the SSD increases by a certain amount of time; vice versa, when (s)he is not able to suppress the response, the SSD decreases by the same amount of time. This tracking procedure of the SSD, leads to around 50% of successful Stops and it is necessary to discourage participants to adopt waiting strategies when they perform the task. According to the authors of the model, the earliest SSD should be close to zero, while the latest SSD should be a little bit longer than the mean Go signal RT (Logan, 1994). Please, see Table 1 for a summary of the most important SST’s indexes.

**Table 1:** List of the main indexes of the Stop Signal Paradigm

Index	Definition	Functional meaning
Stop Signal Delay (SSD)	The delay between the presentation of the go stimulus and the Stop-Signal. It is dynamically adjusted according to participant’s performance	It allows to (i) obtain around 50% of successful Stops; (ii) discourage participants to adopt waiting strategies during the task; and (iii) estimate the SSRT
p(respond|signal)	Probability of responding on a Stop trial. It should be relatively close to 0.50	It allows to (i) estimate the SSRT; and (ii) evaluate whether the paradigm has been properly designed
Stop-Signal Reaction Time (SSRT)	It is a covert latency underlying the Stop process, which is estimated using the race model	It represents an unobservable amount of time needed to stop a movement after the presentation of a Stop-Signal

To the best of our knowledge, with the exception of Beltrán et al. (2018), no other research on language processing has been carried out exploiting the Stop-Signal paradigm to investigate linguistic negation. Beltrán et al. (2018), by coupling this paradigm with electroencephalographic (EEG) recordings, investigated whether the sentential negation affects the event-related complex with respect to the one recorded when affirmative sentences were presented. To this end, Beltrán et al. (2018) used Spanish hand-action-related sentences, expressed in the second singular person of the future tense (e.g. “Now [yes] you will cut the bread”, “Now you will not cut the bread). The experimental design was constituted by 19 practice trials followed by three experimental blocks (each containing 96 experimental and 16 filler sentences). The ratio between the Go- and Stop-tasks was 1:1, given that the Stop-Signal was present in 50% of the trials. The staircase procedure started from 150 ms, and it was adjusted increasing or decreasing by 50 ms (100–400). In ~ 44% of the cases, trials were followed by a yes/no recognition task, with the aim to maintain participant’s attention. Behavioural results showed that participants were faster to inhibit their responses during the presentation of affirmative than negative sentences. Besides, EEG data demonstrated that (i) the amplitude of N1 and P3 (both known to be involved in the inhibition mechanisms, e.g., Kenemans, 2015) were enhanced by successful inhibition, and (ii) N1 amplitude was higher for successful inhibitions of negative sentences, showing an effect of sentence polarity. Through the source analysis, the authors identified the Right Inferior Frontal Gyrus (rIFG) as the source of the N1.

Despite its novelty, this study has a few methodological limits. First, the frequency of Stop-Signal occurrences was high (50%), affecting the participants’ level of attention and the probability of adopting proactive strategies. Second, the sample size was dramatically decreased. They started from 28 participants (23 female), ending up with 15 participants because the other 13 participants had a poor behavioural performance with the Stop-Signal task: they did not succeed in 55% of inhibition (44.5–68%).

Thus, the present study has two main aims: (i) to verify whether negative sentences recruit inhibition resources and whether this interaction between language and motor inhibition mechanisms leads to shorter or longer SSRT than affirmative sentences, (ii) to clarify whether the SST could be considered a good paradigm to assess this issue. Considering the existing literature on the processing of sentence negation suggesting that negative sentences have longer processing times compared to their affirmative counterparts (Carpenter & Just, 1975; Clark & Chase, 1972; Kaup, 2001; Kaup & Zwaan, 2003; MacDonald & Just, 1989), and in the light of previous results on the language–action interaction, we assume that the interference effect is more likely than the facilitation one. In fact, the longer processing time for negative sentences might lead, in our experimental paradigm, to an overlap between the linguistic and the motor task, which both require the same cognitive resources (i.e., the inhibitory mechanisms). In other words, both the processing of sentence negation and the inhibition of a pre-planned movement will compete for inhibitory resources. This will determine interference between these two processes which will result in Negative SSRTs higher than Affirmative SSRTs.

## Materials and methods

### Participants

Thirty healthy young adults (15 females, M_age_ = 27.87, SD_age_ = 3.23, range_age_ = 23–35) took part in the study. Taking into account the high percentage of participants removed from analyses by Beltrán et al. (2018), the number of participants exceeded the a priori required total sample size (*n* = 27) estimated by means of statistical power analysis (a priori sample size *n*. evaluated for one-tailed *t* test: *α* = 0.05, 1 − *β* = 0.80, effect size = 0.5; G*Power 3.1.9.4; Faul et al., 2009). Inclusion criteria were (i) age range from 18 to 35 years old; (ii) right-hand dominance, as assessed by the Edinburgh Handedness Inventory (Oldfield, 1971); (iii) Italian as mother tongue; (iv) absence of learning disabilities or other language impairments; (v) normal or corrected-to-normal visual acuity; and (vi) absence of psychiatric and neurological diseases. Five participants were discarded from data analyses due to poor compliance with the SST: one of them obtained a high probability to respond to signal in the ^n^ Negative condition (66%), while the others made more than 6.5% omission errors in the Go-task (6.72%, 9.38%, 17.03% and 12.81%, respectively). Moreover, two more participants were excluded due to a poor performance in the recognition task (mean accuracy < 70%). In conclusion, our final sample was composed by twenty-three healthy subjects (13 female; M_age_ = 28.43, SD_age_ = 3.27, range_age_ = 23–35). Since we excluded seven participants, we estimated a posteriori the power of our analysis with a results of 0.60 (one-tailed paired *t* test: effect size = 0.41, *α* = 0.05, sample size = 23; G*Power 3.1.9.4; Faul et al., 2009).

All participants provided a written informed consent to participate in the study, which was approved by the local ethical committee “Comitato Etico dell’Area Vasta Emilia Nord” Institutional and was conducted in accordance with the Declaration of Helsinki 2013.

### Stimuli

The stimuli consisted of ten hand-action-related sentences selected from a larger sample. Specifically, we first created one set of verbs constituted by 20 hand-action/concrete verbs and 20 abstract verbs, balanced for frequency of use (Bambini & Trevisan, 2012), number of syllables and characters. Then, we produced 40 two-word sentences, anticipating the first-person subject to the verbs. An independent group of twenty-four participants (12 female, M_age_ = 35.42, SD_age_ = 13.37, range_age_ = 20–69) took part in a validation study aimed at selecting ten hand-action-related sentences and ten abstract sentences. Since the focus of the validation was about the verb-related meaning, all the sentences were presented in affirmative polarity (i.e. “Io scrivo”/ “Io ricordo”, Italian version of “I write”/ “I remember”, respectively). For more information about the stimuli validation, see ESM_1 in Online Resources.

Given our aims, which included an assessment for the Stop-Signal Paradigm’s suitability, and considering that the experiment was very long (about 90 min), in the present study, only concrete sentences, both in affirmative and negative polarity, were included. It is important to emphasise that Italian is a pro-drop language, so that the subject may be omitted. In this way, each sentence of the task is constituted by two words. The whole list of selected items is reported in Table 2.

**Table 2:** List of all the experimental stimuli

Affirmative	Negative
Io gratto *(I scratch)*	Non gratto *(I don’t scratch)*
Io scolpisco *(I sculpt)*	Non scolpisco *(I don’t sculpt)*
Io disegno *(I draw)*	Non disegno *(I don’t draw)*
Io firmo *(I sign)*	Non firmo *(I don’t sign)*
Io mescolo *(I mix*)	Non mescolo *(I don’t mix)*
Io pulisco *(I clean)*	Non pulisco *(I don’t clean)*
Io raccolgo *(I pick up)*	Non raccolgo *(I don’t pick up)*
Io tocco *(I touch)*	Non tocco *(I don’t touch)*
Io scrivo *(I write)*	Non scrivo *(I don’t write)*
Io prendo *(I take)*	Non prendo *(I don’t take)*

In italics the English translation of the sentence

Differently from Beltrán et al. (2018), we employed very short sentences for two main reasons: the first one is the attempt to help participants to focus their attention only on the relevant aspects of the experimental trial (polarity and verb), while the second one is the need to shorten as much as possible the duration of the task (see Section "Experimental design"). Furthermore, whereas, Beltrán et al. (2018) used the second person singular of the simple future tense, we used the first person singular of the present tense. The first person’s choice was based on results from the literature suggesting that an interference effect is more likely to be obtained with the first and third person singular compared to the second person singular (for a discussion, see Borghi, 2012). Finally, we chose not to include filler sentences because they were useless to the present study’s aims.

### Experimental design

The twenty Affirmative and Negative selected sentences (see ESM_1 in the Online Resources for more information about stimuli validation) were repeated 48 times each for 960 trials. Of these, 33% were Stop trials (half Affirmative), and 67% were Go trials (half Affirmative). The number of trials was established considering that the Stop-Signal Paradigm’s dynamic structure should produce about 50% of successful inhibition and the desire to obtain about 80 items for each experimental condition (successful and unsuccessful stop trials, UST).

We maintained the same trial structure of Beltrán et al. (2018) presenting the sentences word by word at the centre of the screen. Words were written in black capital letters (Arial font size 48 points) on grey background, and they were interposed with 200 ms empty grey screens. Each Go trial started with a 500 ms fixation cross, followed by the polarity-referred word (i.e., “Io” for Affirmative condition and “Non” for Negative condition), which lasted 250 ms. As for the Go-task the verb was, presented first, of random duration between 250 and 900 ms, to avoid possible anticipation errors in its perception, followed by a left- or right-pointing arrow (Go stimulus) appearing on it. At the appearance of the Go stimulus, participants were instructed to respond as quickly and accurately as possible to its direction using the mouse with their right hand, pressing the right- or the left-button for the right- or left-pointing arrow, respectively. Participants had 600 ms to respond to the arrow presentation. When this time has expired, the answer was considered missing. The correct execution of the Go-task was marked by a 250 ms positive feedback at the centre of the screen, and the trial conclusion was determined by a 1000 ms inter-trial interval (ITI).

The Stop trial structure was similar to the Go one, but a 100 ms auditory Stop-Signal followed the Go stimulus. A staircase procedure established the delay between the onset of Go stimulus and Stop-Signal onset (i.e., SSD). The initial SSD was set at 132 ms (8 refresh rate of our 60 Hz screen), and it was dynamically adjusted as a result of participant’s performance: it increased and decreased based on the participant’s ability or inability to withhold his/her response to the arrow, respectively. The SSD ranged from 33 to 495 ms (30 refresh rate). Differently from Beltrán and colleagues (2018), who modulated the staircase in steps of 50 ms, we modified the SSD in steps of 33 ms to obtain greater modulation and sensitivity. Participants were instructed to try to withhold their response to the Go-task. Specifically, taking into account the difficulty in doing that, we told them not to worry if they made mistakes, as it was expected that they would not always be able to stop. Also for the Stop trials, the erroneous responses (i.e., UST) were registered within 600 ms from the onset of the Go stimulus.

**Fig. 1:**
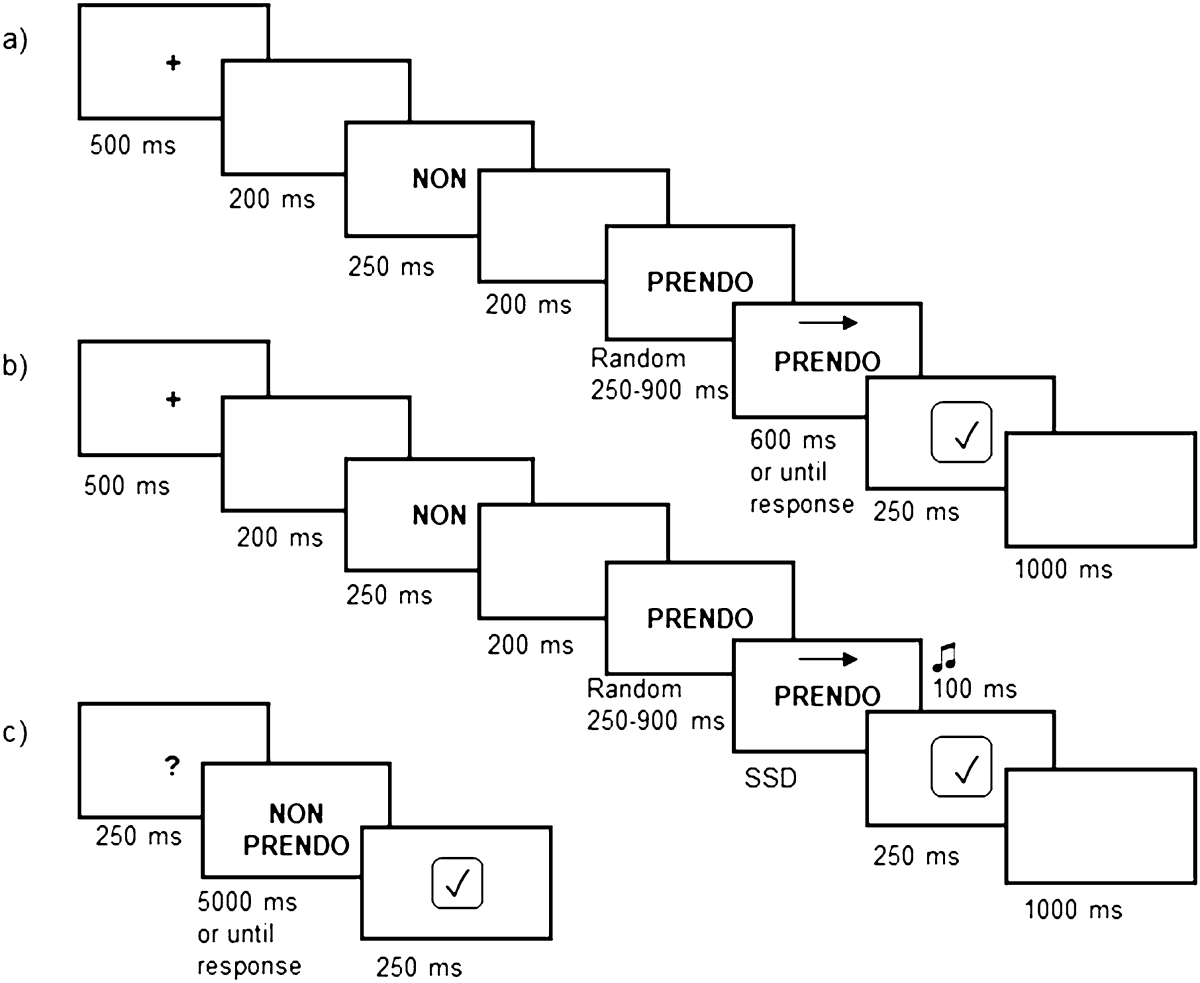
The experimental design. Italian two-word sentences were presented word by word at the centre of the screen. Participants were instructed to perform the SST, which is constituted by two tasks. The first one (i.e., the Go-task, panel **a**) was to answer as quickly and accurately as possible to the presentation of the arrow (Go stimulus), pressing with the right hand the left or the right mouse button according to its direction. In the second one (i.e., the Stop-task, panel **b**), participants were instructed to refrain from responding when an auditory Stop-Signal followed the Go signal after a variable Stop Signal Delay (SSD). Indeed, the onset of the auditory Stop-Signal depends on participants’ performance, increasing or decreasing by 33 ms according to their ability to successfully or unsuccessfully inhibit their response, respectively. 120 trials (~ 12.5%) were followed by a yes/no recognition task (panel **c**)

In 12.5% of the total trials sample, the ITI was followed by a yes/no recognition task. It was anticipated by a question mark lasting 250 ms. It was constituted by a two-word sentence presented for 5000 ms: participants had to determine whether it was identical or different from the one presented in the experimental trial (see Fig. 1 for an exemplification of the experimental design). Participants were instructed to press indifferently one of the mouse buttons only when the sentences were similar, i.e., when they had both the same polarity and the same verb. In 50% of the recognition tasks, the sentences were “similar”, while in the remaining 50%, they were “different” (25% in the polarity and 25% in the verb). The correct execution of the recognition task was marked by a 250 ms positive feedback shown at the centre of the screen. The catch trials were included with a dual purpose: (i) to keep participants’ attention; and (ii) to ensure that they read the sentences. The experimental paradigm was created and controlled using E-prime software (version 2.10; Psychology Software Tools, Inc.).

The 960 experimental trials were presented in seven blocks (containing ~ 137 trials each) lasting 8.5 min each, allowing participants to rest between one block and another. In each block, the proportion between Go and Stop trials was maintained (2:1, respectively), and it was balanced by polarity and direction of the arrow.

Before starting the experiment, participants carried out a training session consisting of two phases. To have control on participants’ RTs, the first part was constituted by 48 Go trials. To make participants familiar with the experimental task, the second part was constituted by both Go and Stop trials (Go = 92, Stop = 46; 18 trials were followed by the yes/no recognition task). All the training trials included two-word sentences not selected for the experimental task to avoid familiarization effects.

Participants were comfortably seated in a quiet room, with about 60 cm from the screen. The experimenter was present in the room only during the training session, to ensure that tasks were clearly understood.

As a final note, two more aspects make our experimental design different from Beltrán et al. (2018) study: (1) we instructed participants to respond only with their right-hand, and (2) we inserted a 1000 ms grey ITI after the trial, allowing the return to baseline of the brain activity. The EEG study of Beltrán et al. (2018) did not include a blank ITI. In Table 3, we reported all the differences between the two experimental designs.

**Table 3:** Methodological differences between Beltrán et al. (2018) and the present study

Features	Beltrán et al. (2018) experiment	The present experiment
Stimuli		
Structure	Long: five-word sentences	Short: two-word sentences
Verb tense	Future	Present
Perspective	Second person	First person
Total amount	288 (72 Go Aff, 72 Go Neg, 72 Stop Aff and 72 Stop Neg)	960 (320 Go Aff, 320 Go Neg, 160 Stop Aff and 160 Stop Neg)
Filler sentences	Included, 48	Not included
Verb duration	700 ms	Random between 250 and 900 ms
Frequency of the Stop-Signal	50%	33%
Staircase	From 150 ms; increase/decrease in step of 50 ms	From 132 ms; increase/decrease in step of 33 ms
Mean SSD	Estimated by the classical mean	Mid-run estimate method
ITI	–	1000 ms
Recognition task	In 44% of trials	In 12.5% of trials
Training	Short training constituted by 19 practice trials. Data not analysed	Two-phases training. The first one was constituted by 48 trials and was included to measure “baseline” RTs in Go trials; the second one included 137 trials and was used to familiarise with the SST. It was not analysed
Response modality	Using a gamepad with both hands	Using a mouse with the right-hand
Positive feedback	Not included	Included

### Analysis

For each participant and separately for Affirmative and Negative sentences, we calculated mean Go RT, mean UST RT, mean SSD, p(response|signal), SSRT, and error percentages. Indeed, we chose to exclude all the trials containing errors committed by the participants: (i) anticipations, when participants pressed the mouse before the onset of the Go stimulus (i.e., the arrow); (ii) omissions, when participants did not respond to Go stimuli in Go trials; and (iii) direction errors, when participants erroneously pressed the right button while a left-pointing arrow was presented on the screen (and vice versa). See Table 4 for more information about the percentage of errors.

**Table 4:** Mean percentage of each type of error in the sample of 23 participants

Type of errors	Mean percentage
Anticipation on Go and Stop trials	0.08
Choice on Go and Stop trials	1.16
Omission on Go trials	3.36

As for the computation of mean SSD, the staircase is constituted by a sequence of SSDs that increase or decrease according to the participant’s performance (see Section "Experimental design" for more information about the tracking procedure), creating ascending and descending runs of value. To calculate the mean SSD, we used the mid-run estimates method, according to which the mean value should be estimated by averaging the SSDs from the midpoints of every second run (Levitt, 1971; Wetherill & Levitt, 1965). Unlike the arithmetic mean used by Beltrán et al. (2018), this method is more suitable for staircase procedure.

Finally, to estimate SSRT values, we chose the integration method with the exclusion of omissions in Go trials. Therefore, separately for Affirmative and Negative sentences, the distribution of the RTs of the Go trials was rank ordered and the *n*th reaction time was identified, where *n* is the value obtained by the number of RTs that constitute the RT distribution multiplied by the probability to respond to the signal in the Stop trials of the same condition. The SSRT was then calculated subtracting the mean SSD of the same condition from the Go RT obtained before (Logan et al., 1984; Matzke et al., 2018; Verbruggen et al., 2019). As an example, if we have 200 Affirmative Go trials and a p(respond|signal) in the Affirmative condition of 0.45. The distribution of the RTs of the 200 Go trials should be rank ordered with the aim to identify the *n*th fastest reaction time of the distribution (i.e., the Go RT), which, in our example, is the 90th (200 × 0.45 = 90). If the Go RT is 364.00 ms and our mean SSD is 169.22, we can estimate the Affirmative SSRT for this participant subtracting the mean SSD from Go RT (364.00 ms–169.22 ms = 194,78 ms). Please, see ESM_3 of the Online Resources for a complete overview of the data.

Since not all the variables were normally distributed after the base-10 logarithmic transformation, as assessed by Shapiro–Wilk normality tests (in ESM_2 of the Online Resources are reported the values pre- and post-log10 transformation), we used both parametric and non-parametric tests for the statistical analysis. To test our main hypothesis on the SSRTs (Affirmative vs. Negative), we used a one-tailed paired *t* test, since we had a precise hypothesis about the direction of the effect (i.e., Negative SSRT higher than Affirmative SSRT). We also investigated the effect of polarity between Go- and UST-RTs by means of a non-parametric Wald-type statistic (WTS) for Repeated Measures Data in within Factorial Designs with two within-factors: Polarity (two levels: Affirmative and Negative) and Condition (two levels: Go and UST). This non-parametrical rank-based test was elaborated to analyse longitudinal data in factorial designs with the aim to investigate treatment effects, time effect and their interaction effects. Indeed, this analysis estimates the relative treatment effects (RTEs), comparable to the concept of relative marginal effects (for more information about the analysis and the nparLD R package, see Noguchi et al., 2012; for a similar application of WTS on repeated measures data see Soto et al., 2020; Sykownik & Masuch, 2020). However, repeated measures designs are conceptually included in longitudinal designs, since one of their characteristics is the inclusion of several measurements of the same variable collected on the same participant.

Furthermore, we checked whether, increasing the probability to inhibit the Stop-Signal, participants slowed their RTs in the experimental session with respect to the first training block. To evaluate this aspect, we used a non-parametric Wald Test for Repeated Measures Data in within Factorial Designs with two within-factors: Polarity (two levels, Affirmative and Negative) and Go-RTs (two levels: Training and Experimental).

We also investigated whether a successful or failed Stop trial induced a “procrastination strategy” in the subsequent Go trial (Mirabella et al., 2006; Mirabella et al., 2012a; Verbruggen & Logan, 2009). In particular, we hypothesised that successful Stop trial leads to longer RT in the subsequent Go trial and this response slowing should be more marked in Negative Go trials, since we assume that during their processing there is the involvement of inhibitory mechanisms that lead to higher RTs. To explore the after-effect, we ran a non-parametric Wald Test for Repeated Measures Data in within Factorial Designs with two within-factors: StopType (two levels, Failed and Successful) and TargetGo (two levels: Affirmative and Negative). To address this issue comprehensively, we also investigated whether the RT of a Go trial was modulated by its polarity and the immediately preceding Go trial’s polarity. For this reason, we performed a parametric Analysis Of the Variance (ANOVA) with two within-factors: PreviousGo (two levels, Affirmative and Negative) and TargetGo (two levels: Affirmative and Negative).

Finally, we performed a non-parametric McNemar test with the purpose of investigating the effect of polarity in the performing of the different types of errors. However, we excluded anticipation errors from the analysis due to their low percentage in the total trial sample (see Table 4).

Data from the first training phase, and the whole experimental session were analysed using MATLAB (version 2019a) and R Studio (version 1.2.5033; R Core Team, 2019).

## Results

**Fig. 2:**
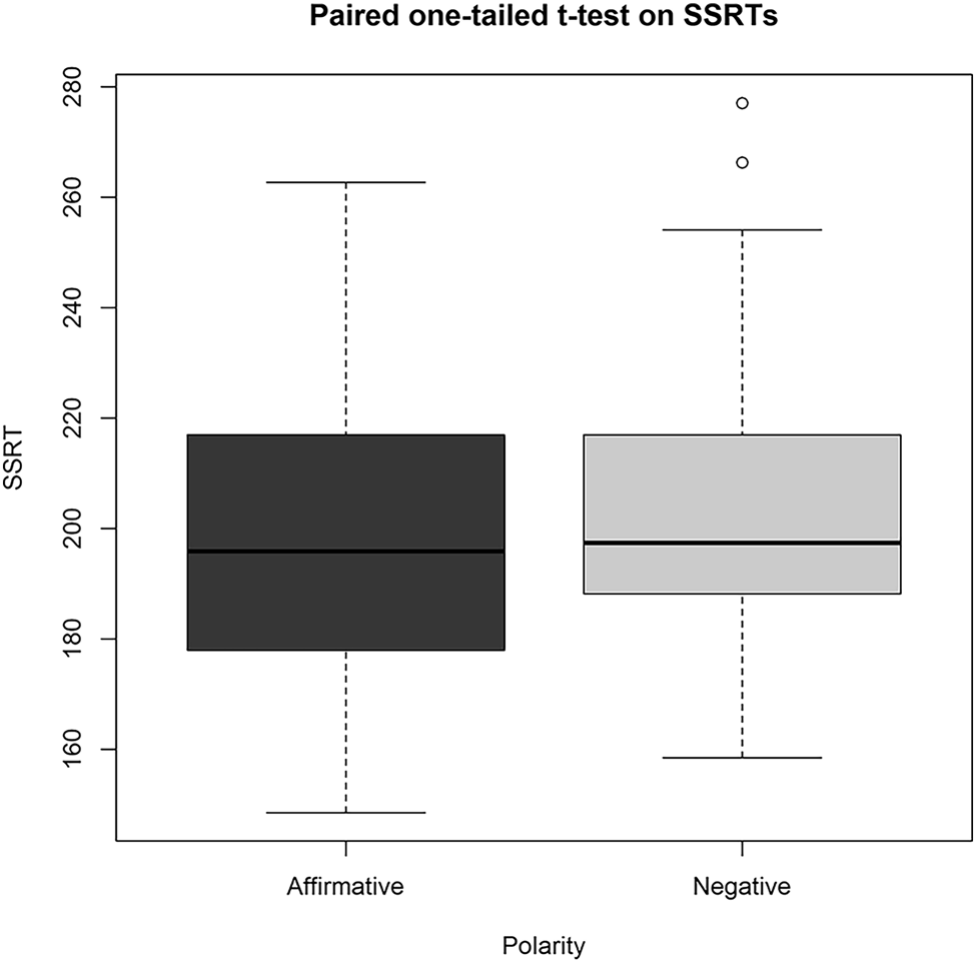
*T* test analysis boxplot. As shown in the graph, Negative Sentences (Mdn = 197.42) present SSRT longer than the Affirmative ones (Mdn = 195.85). Error bars represent the first and fifth quartiles of the mean. The two circles in the graph represent outlier SSRT values. However, since SSRT is an estimated value and since the raw data of these two participants were not outlier, we include them in the analysis

The paired one-tailed *t* test on the SSRTs (see Fig. 2) showed a significant effect of Polarity (*t* (22) = − 2.11; *p* = 0.02, *r* = 0.41): Affirmative SSRTs (*M* = 200.67 ms, SD = 30.27) were significantly faster than Negative SSRTs (*M* = 207.31 ms, SD = 30.69). Since this was the main analysis of our project, for the sake of clarity, we chose to use not-transformed data for the descriptive values and graphs.

**Fig. 3:**
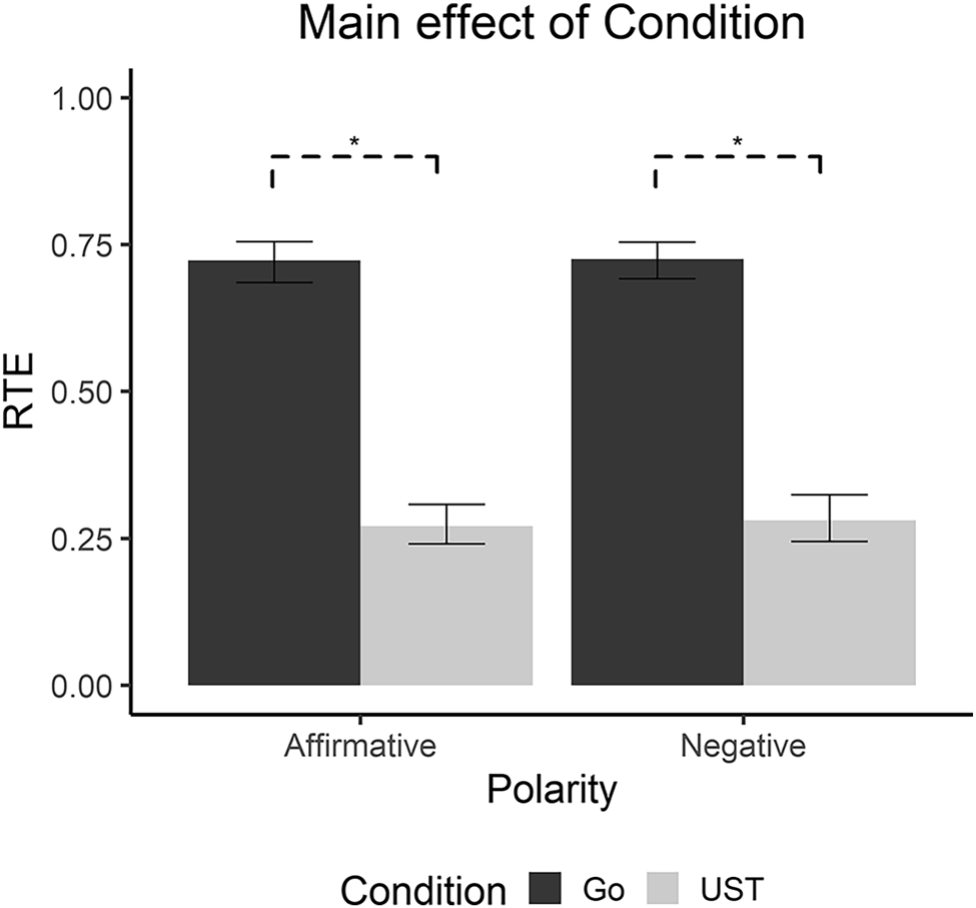
Main effect of condition. As shown in the graph, both Negative and Affirmative sentences present slower RTs in the UST Condition. Error bars represent standard confidence intervals.*p < 0.001

The Wald Test for Repeated Measures Data in within Factorial Designs on Go-RT and UST-RT showed only the significance of the main effect of Condition (*W*_(1, 22)_ = 652.12, *p* < 0.001): for both Affirmative and Negative sentences, Go RTs (Affirmative: Rank Mean = 67.00, RTE = 0.72, CI 0.68–0.75; Negative: Rank Mean = 67.17, RTE = 0.72, CI 0.69–0.75) were higher than UST RTs (Affirmative: Rank Mean = 25.48, RTE = 0.27, CI 0.24–0.31; Negative: Rank Mean = 26.35, RTE = 0.28, CI 0.24–0.32) (See Fig. 3).

The same non-parametric Wald Test, for Repeated Measures Data in within Factorial Designs on the GO RTs, did not reveal significant effects, suggesting that there were no significant differences on the RTs between Training (Affirmative: Rank mean = 49.67, RTE = 0.53, CI 0.45–0.62; Negative: Rank mean = 52.63, RTE = 0.57, CI 0.48–0.64) and Experimental phase (Affirmative: Rank mean = 42.17, RTE = 0.45, CI 0.38–0.53; Negative: Rank mean = 41.52, RTE = 0.45, CI 0.37–0.52).

As for the after-effect analysis, both the non-parametric Wald Test for Repeated Measures Data in within Factorial Designs and the parametric ANOVA on the GO RTs did not reveal significant effects. As for the effect of Stop trial on the subsequent Go trial, results show that there were no significant differences on the Go-RTs according to their previous Stop trial and their Polarity (Affirmative Fail: Rank Mean = 46.65, RTE = 0.50, CI 0.44–0.57; Negative Fail: Rank Mean = 44.91, RTE = 0.48, CI 0.42–0.54; Affirmative Successful: Rank Mean = 46.65, RTE = 0.50, CI 0.46–0.55; Negative Successful: Rank Mean = 47.78, RTE = 0.51, CI 0.45–0.58). Regarding the effect of previous Go trial polarity and the target Go trial polarity on RTs, the analysis showed no significant differences on the Go-RTs (Mean Affirmative-Affirmative = 376.14 ms; Mean Affirmative-Negative = 372.89; Mean Negative-Affirmative = 376.49; Mean Negative-Negative = 375.92).

**Fig. 4:**
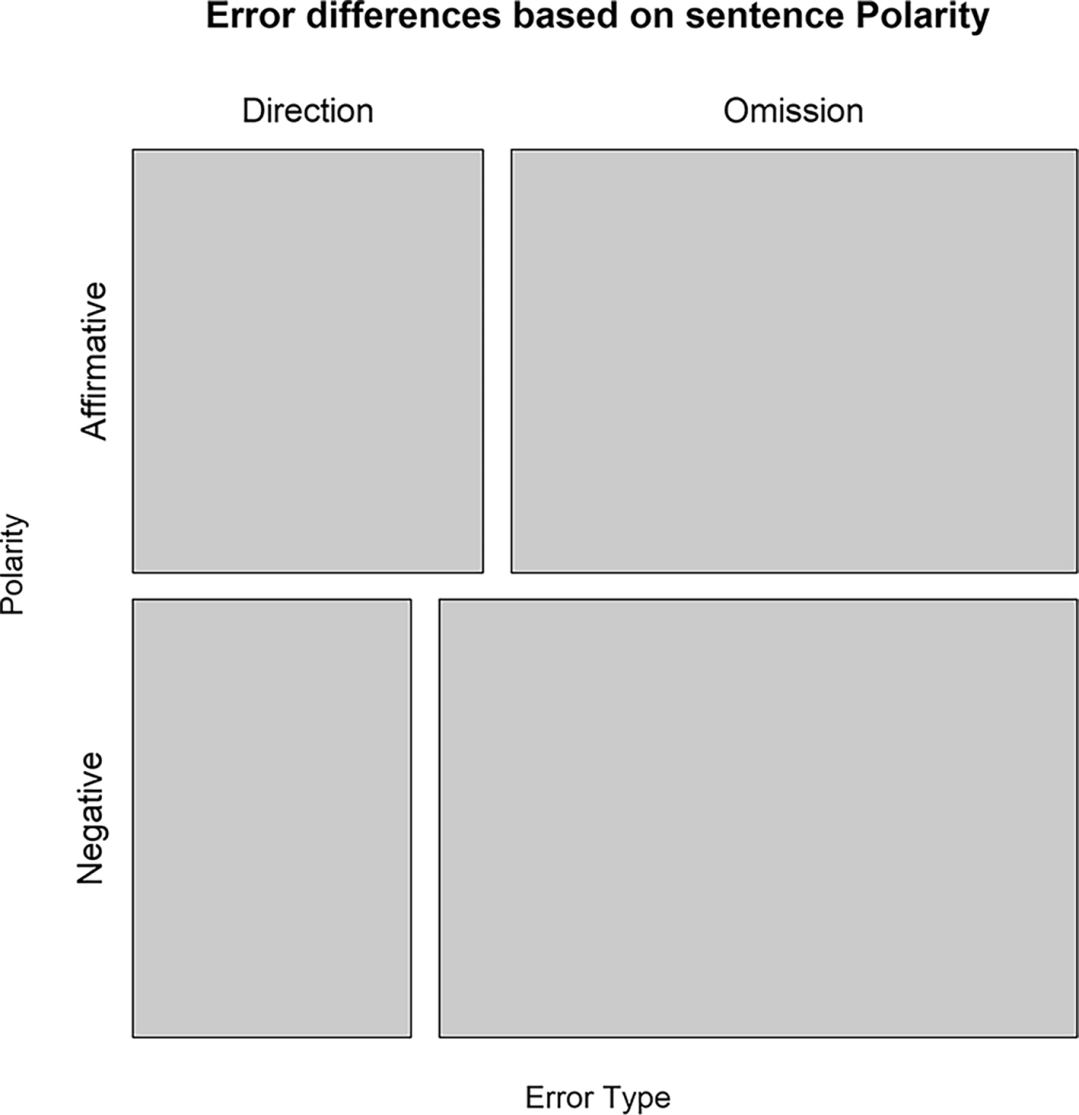
Mosaic plot on the effect of polarity on error type

Finally, the non-parametric McNemar test on the Error showed a significant association between error types and sentence polarity (*x*^2^_(1)_ = 35.82, *p* < 0.001). Contingent table showed that Direction errors were more frequent in the Affirmative condition (141) respect to the Negative one (116), while the opposite pattern is showed for Omission errors (Affirmative = 228; Negative = 266) (see Fig. 4).

## Discussion

The present study shows that Negative sentences have longer SSRTs than their Affirmative counterparts. Furthermore, our results also revealed two different patterns for each type of error committed by participants. Direction errors were more frequent in Affirmative sentences than in the Negative ones. In contrast, a greater amount of omission errors was found in Negative sentences with respect to the Affirmative ones. The higher rate of omission errors in Negative sentences reflects the involvement of inhibitory mechanisms. Thus, the present study supports the hypothesis that the processing of sentential negation recruits motor inhibition mechanisms.

According to the existing literature, the SSRT of hand/arm-movements in healthy adults is around 200–250 ms (Mirabella et al., 2006; Ramautar et al., 2004). To make sure that participants did not perform the task using proactive-waiting strategies during the experimental sessions, we compared their RTs with those obtained in the first training block (i.e., the one containing only Go trials). Results showed that the RTs in the two experimental blocks did not differ significantly, making our results more reliable.

Thus, our data confirmed findings from Beltrán and colleagues (2018). The recruitment of the mechanisms for motor inhibition in sentential negation has also been investigated in a small set of studies which exploited the GO/NOGO paradigm (Beltrán et al., 2019; Liu et al., 2019; de Vega et al., 2016) during the silent reading of sentences (Foroni & Semin, 2013; Papeo et al., 2016), and the typing of hand-action-related verbs (García-Marco et al., 2019). Findings from these studies are not directly comparable with our data, because coming from a different experimental paradigm that might reflect the contribution of a different mechanism for motor inhibition (Littmann & Takács, 2017; Raud et al., 2020). However, they all highlight the involvement of inhibitory resources in the processing of Negative sentences. Our results contribute to this picture, showing that the processing of sentential negation recruits mechanisms for motor inhibition and, thus, provides support to the embodied account of language processing even for abstract aspects of language, like the negation logic operator.

Furthermore, as for the analysis of RTs in Go trials and UST, our findings showed only a main effect of Condition, which confirmed the independence assumption. However, it is interesting to note that we did not find a main effect of Polarity, whereas a significant difference based on sentence polarity, with Negative RTs longer than the Affirmative ones, could have been expected according to previous results showing that negative sentences have longer processing times than their positive counterparts. Longer RTs in the processing of negative sentences have been accounted for by cognitive effects such as (i) reduced accessibility of the negated concept (Kaup, 2001; Kaup & Zwaan, 2003; MacDonald & Just, 1989); (ii) the elicitation of a complementary scenario (Kaup et al., 2005; Orenes et al., 2014); (iii) an increase of the cognitive effort (Carpenter & Just, 1975; Clark & Chase, 1972; Kaup et al., 2006). In all these cases, the effect is reflected in longer RTs during the processing of Negative sentences compared to the Affirmative ones. Our results did not replicate these findings, since we did not find any effect of polarity between RTs in Go trial and UST. However, it is important to note that our findings are not in contrast with these previous results. In fact, differently from these latter studies, which aimed to investigate the processing of sentence negation, the present study had the aim to investigate whether motor inhibition mechanisms underpin the processing of negation. To this purpose, we applied the SST, a paradigm for the research on inhibitory mechanisms, to the study of language and sentence negation.

With regard to this experimental paradigm, there is evidence that a successful Stop trial could activate inhibitory processes, leading to effects in the subsequent Go-task (inhibitory after-effect: Anguera et al., 2013; Rieger & Gauggel, 1999; Verbruggen et al., 2004). This effect might induce slower RTs making our data not directly comparable with previous studies. Moreover, the inhibitory after-effect in our study could have been further modulated by sentence polarity in two ways: we hypothesised that (i) a successful stop trial might lead to longer RT in the subsequent Go trial, and this response slowing might be more marked in Negative Go trials; and (ii) the RT of a Go trial could have been modulated by its polarity and the polarity of the immediately preceding Go trial, with longer RTs when a Negative Go trial precedes a Negative Go trial. However, our results didn’t show any significant effects on both these hypotheses.

Finally, we focussed our attention on the error type committed by participants. Our results revealed two different patterns for the two considered types of error, with a greater amount of direction errors in Affirmative sentences than in the Negative ones. In contrast, omission errors were more frequent in Negative sentences with respect to the Affirmative ones. The differences in polarity based on error type supported the involvement of inhibitory mechanisms underpinning the processing of sentence negation. Indeed, the presence of greater omission errors in Negative sentences reflected the involvement of inhibitory mechanisms, while this did not occur in the Affirmative polarity sentences.

Finally, the present study also aimed to assess whether the SST can be considered a good tool to explore the involvement of inhibition resources in negation processing. It is now time to discuss our results about this second point.

Beltrán and colleagues were the first to use the Stop-Signal paradigm to investigate the inhibitory aspects underlying sentence negation (see Beltrán et al., 2018). However, despite their methodological innovation and although our study confirmed their results (Negative SSRT higher than Affirmative SSRT, *p* < 0.02), we believe that their experimental design has some methodological features that can potentially affect their findings. In particular, we have identified as the three main features: (i) the frequency of the Stop-Signal (50%), which can affect the response of participants, slowing their movements; (ii) the dramatic decrement of the sample size due to poor compliance with the SST, leading to more than 55% unsuccessful stop trial (44.5–68%); and (iii) the use of long sentences, which include the noun to which the verb refers, potentially inducing a context effect.

As Beltrán et al. (2018, 2019) being confident of the great potential of this paradigm, we decided to replicate their experiment by making some key changes. In the present experiment, we excluded only seven participants from the original sample of thirty, due to poor compliance with the paradigm (i.e., five participants) and due to low accuracy in the recognition task (i.e., two participants), allowing us to perform analysis on larger sample size.

However, we are aware that also our study has some limitations that could affect the results. First, despite the division of the experimental task into blocks, the excessive task duration could affect participants’ attention and performance. Furthermore, the interval of time between the disappearing of polarity-related word and Go-stimulus presentation varies randomly from 450 to 1150 ms. Even though we found a significant difference between SSRTs according to the polarity, we guess that such long time interval could have mitigated this effect of interest. We believe that this extremely longer interval of time could also be the reason for the absence of significant results in the after-effect analysis.

In conclusion, the present study shows that: (i) inhibitory mechanisms are involved in the processing of language negation, with respect to the affirmative counterpart, (ii) reflecting the presence of an interference effect, instead of a facilitation one, and (iii) this involvement is demonstrated through the use of the SST, which allows to estimate the SSRT. It follows that the SSRT can be considered, together with the RTs and the error rate, a useful index to investigate the involvement of motor inhibitory resources in the processing of sentence negation.

## Supplementary Information

Below is the link to the electronic supplementary material.

Supplementary file1 (DOCX 35 KB)

## Data Availability

The datasets analysed during the current study are available from the corresponding author on reasonable request.
