# Supplementary material for: Is motor inhibition involved in the processing of sentential negation? An assessment via the Stop-Signal Task
Source: Psychol Res. 2021 Apr 27;87(1):339–52. doi: 10.1007/s00426-021-01512-7 (PMC9873753; doi:10.1007/s00426-021-01512-7)
Supplement: Supplementary file 1 — Supplementary file1 (DOCX 35 KB) [file 426_2021_1512_MOESM1_ESM.docx]

**Is motor inhibition involved in the processing of sentential negation?**

**An assessment via the Stop Signal Task**

Montalti, M.^1*^, Calbi, M. ^1^, Cuccio, V.^2^, Umiltà, M.A.^3^, Gallese, V. ^1,4^

^1^ Department of Medicine and Surgery – Unit of Neuroscience, University of Parma, Parma, Italy

^2^ Department of Cognitive, Psychological, Pedagogical Sciences and Cultural Studies, University of Messina, Messina, Italy

^3^ Department of Food and Drug, University of Parma, Parma, Italy

^4^ Berlin School of Mind and Brain, Humboldt-Universität zu Berlin, Germany

*Corresponding author

Department of Medicine and Surgery, Unit of Neuroscience, University of Parma, Parma, Italy

E-mail: [marti.montalti@gmail.com](mailto:marti.montalti@gmail.com)

[martina.montalti@unipr.it](mailto:martina.montalti@unipr.it)

***Journal***: Psychological Research

**ESM_1** – Stimuli validation.

We created two pseudo-randomized online surveys (Sondaggio-Online, https://www.sondaggio-online.com), in which participants were asked to rate each sentence using a 1 to 7 Likert scale: 1) How fast is the sentence in evoking a mental image, a visual representation, a sound or other perceptual experiences?; 2) How much movement is *implied by the sentence?*; 3) *How much abstract or concrete do you judge the sentence?* (Half of the participants evaluated a reverse question: *How much concrete or abstract do you judge the sentence?*)*; 4) How is the valence of the sentence?; 5) How would you rate the arousal of the sentence?; 6)How much do you think the sentence expresses an involvement of the hand?.* Question 1) and 2) were taken from the stimuli validation of Liuzza and colleagues (2011). The mean value of each sentence question 1), 2) and 3) were summed, with the aim to choose the concrete sentences with the highest scores and the abstract sentences with the lower scores. The same procedure was also applied for question number 6), but we considered these rates just in a qualitative way, comparing the first ten items with the first ten obtained in the previous step. Then, the arousal and valence scores of the first ten items were analysed by means of non-parametrical Wilcoxon signed-rank Test to check whether the two abstract and concrete sub-sets were significantly different, showing no significant results (arousal: z = -0.29, *p* = .98, r = -0.06; valence: z = -0.86; *p* = .93, r = -0.18). Finally, a Mann-Whitney Test applied to check whether the two categories were still balanced for frequency of use (U = 27.50, z = -1.9, p = .09, r = -0.39), number of syllables (U = 33.50, z = -1.2, p = .22, r =-0.24) and number of characters (U = 31.50, z = -1.5, p = .17, r = -0.31) did not reveal significant effects. Data from the frequency of use were provided by the CoLFIS (Corpus e Lessico di Frequenza dell'Italiano Scritto, Bambini & Trevisan, 2012).

**ESM_2 –** Results of Shapiro-Wilk normality test of mean RTs distributions before and after log10 transformation.

| **Analysis** | **Variable** | **Shapiro-Wilk test**  **(before log10 transformation)** | **Shapiro-Wilk test**  **(after log10 transformation)** |
| --- | --- | --- | --- |
| *Principal Analysis* | *Training Go Affirmative* | W = 0.97, *p* = .67 | W = 0.60, *p* = .00* |
|  | *Training Go Negative* | W = 0.97, *p* = .74 | W = 0.58, *p* = .00* |
|  | *Experimental Go Affirmative* | W = 0.90, *p* = .03* | W = 0.88, *p* = .01* |
|  | *Experimental Go Negative* | W = 0.90, *p* = .02* | W = 0.88, *p* = .01* |
|  | *Experimental UST Affirmative* | W = 0.94, *p* = .19 | W = 0.84, *p* = .00* |
|  | *Experimental UST Negative* | W = 0.96, *p* = .55 | W = 0.79, *p* = .00* |
|  | *Experimental SSRT Affirmative* | W = 0.95, *p* = .29 | W = 0.97, *p* = .68 |
|  | *Experimental SSRT Negative* | W = 0.91, *p* = .04* | W = 0.94, *p* = .15 |
| *After-effect Analysis* | *Go Affirmative preceded by failed Stop* | W = 0.91, *p* = .05 | W = 0.80, *p* = .00* |
|  | *Go Affirmative preceded by successful Stop* | W = 0.95, *p* = .30 | W = 0.66, *p* = .00* |
|  | *Go Negative preceded by failed Stop* | W = 0.88, *p* = .01* | W = 0.89, *p* = .02* |
|  | *Go Negative preceded by successful Stop* | W = 0.91, *p* = .04* | W = 0.65, *p* = .00* |
|  | *Go Affirmative preceded by Go Affirmative* | W = 0.95, *p* = .32 | - |
|  | *Go Affirmative preceded by Go Negative* | W = 0.95, *p* = .35 | - |
|  | *Go Negative preceded by Go Affirmative* | W = 0.96, *p* = .55 | - |
|  | *Go Negative preceded by Go Negative* | W = 0.93, *p* = .10 | - |

**Non-normally distributed variables*

**ESM_3 –** For each participant and for each experimental condition (i.e., independent variable Polarity, 2 levels: Affirmative and Negative) we reported the mean RTs of Go trials and of Unsuccessful Stop Trials (UST), the probability to respond to the signal, the GO RTs, the Stop Signal Delay (SSD), the Stop Signal Reaction Times (SSRT), the accuracy to the recognition task and the percentage of the omission error. Red color identifies participants excluded from the analysis. Five participants were discarded from data analyses due to poor compliance with the Stop Signal Task: one of them obtained a high probability to respond to signal in the negative condition (subject 4), while the others made more than 6.5% omission errors in the go-task (participants 18, 19, 20, 28). Moreover, two more participants were excluded due to a poor performance in the recognition task (mean accuracy < 70%; participants 23 and 26).

| **Participant** | **Mean**  **Go_RT** | | **Mean UST_RT** | | **p(respond\|signal)** | | **GO_RT** | | **SSD** | | **SSRT** | | **Accuracy**  **Recognition Task** | **Omission error %** |
| --- | --- | --- | --- | --- | --- | --- | --- | --- | --- | --- | --- | --- | --- | --- |
|  | **Aff** | **Neg** | **Aff** | **Neg** | **Aff** | **Neg** | **Aff** | **Neg** | **Aff** | **Neg** | **Aff** | **Neg** |  |  |
| 1 | 361.92 | 367.14 | 303.92 | 309.99 | 0.51 | 0.48 | 364.00 | 360.00 | 169.22 | 142.30 | 194.78 | 217.70 | 87.30 | 2.50 |
| 2 | 389.93 | 377.44 | 316.03 | 308.21 | 0.50 | 0.50 | 384.00 | 376.00 | 181.87 | 183.46 | 202.13 | 192.54 | 92.20 | 3.91 |
| 3 | 393.39 | 390.71 | 331.12 | 330.40 | 0.48 | 0.52 | 382.00 | 393.00 | 148.36 | 142.47 | 233.64 | 250.53 | 81.50 | 3.91 |
| 4 | 319.52 | 318.99 | 263.16 | 267.84 | 0.58 | 0.66 | 335.00 | 354.00 | 75.64 | 98.55 | 259.36 | 255.45 | 91.20 | 0.94 |
| 5 | 305.32 | 306.77 | 257.65 | 258.47 | 0.57 | 0.58 | 315.00 | 318.00 | 95.73 | 80.34 | 219.27 | 237.66 | 92.50 | 0.63 |
| 6 | 383.23 | 385.66 | 305.03 | 325.25 | 0.45 | 0.53 | 373.00 | 390.00 | 202.23 | 206.68 | 170.77 | 183.32 | 83.60 | 2.03 |
| 7 | 411.65 | 404.12 | 339.13 | 357.53 | 0.51 | 0.48 | 415.00 | 398.00 | 212.63 | 207.98 | 202.37 | 190.02 | 87.20 | 3.13 |
| 8 | 381.02 | 384.88 | 336.85 | 327.76 | 0.50 | 0.49 | 378.00 | 376.00 | 182.15 | 177.28 | 195.85 | 198.72 | 85.50 | 6.09 |
| 9 | 405.17 | 397.14 | 336.85 | 325.58 | 0.46 | 0.52 | 388.00 | 405.00 | 214.68 | 212.72 | 173.32 | 192.28 | 95.70 | 4.53 |
| 10 | 394.68 | 399.16 | 336.56 | 341.91 | 0.46 | 0.52 | 387.00 | 399.00 | 210.40 | 201.58 | 176.60 | 197.42 | 98.30 | 1.88 |
| 11 | 352.24 | 358.80 | 287.29 | 285.41 | 0.53 | 0.52 | 359.00 | 361.00 | 103.67 | 106.93 | 255.33 | 254.07 | 74.80 | 2.81 |
| 12 | 404.27 | 409.85 | 353.28 | 346.47 | 0.49 | 0.49 | 400.00 | 405.00 | 230.48 | 223.94 | 169.52 | 181.06 | 94.10 | 2.66 |
| 13 | 393.08 | 395.87 | 342.99 | 329.44 | 0.50 | 0.49 | 386.00 | 396.00 | 173.68 | 182.43 | 212.32 | 213.57 | 93.30 | 4.53 |
| 14 | 396.47 | 400.08 | 331.07 | 342.33 | 0.46 | 0.52 | 386.00 | 402.00 | 219.06 | 211.91 | 166.94 | 190.09 | 82.10 | 3.75 |
| 15 | 366.71 | 365.79 | 298.73 | 311.78 | 0.50 | 0.48 | 365.00 | 356.00 | 148.64 | 150.31 | 216.36 | 205.69 | 89.70 | 3.75 |

| **Participant** | **Mean**  **Go_RT** | | **Mean UST_RT** | | **p(respond/signal)** | | **GO_RT** | | **SSD** | | **SSRT** | | **Accuracy**  **Recognition Task** | **Omission error %** |
| --- | --- | --- | --- | --- | --- | --- | --- | --- | --- | --- | --- | --- | --- | --- |
|  | **Aff** | **Neg** | **Aff** | **Neg** | **Aff** | **Neg** | **Aff** | **Neg** | **Aff** | **Neg** | **Aff** | **Neg** |  |  |
| 16 | 420.66 | 415.69 | 354.98 | 368.94 | 0.51 | 0.48 | 427.00 | 411.00 | 223.80 | 224.65 | 203.20 | 186.35 | 96.60 | 4.84 |
| 17 | 414.65 | 416.74 | 362.89 | 363.76 | 0.46 | 0.54 | 409.00 | 426.00 | 260.49 | 251.02 | 148.51 | 174.98 | 94.00 | 6.09 |
| 18 | 363.46 | 368.56 | 304.80 | 307.57 | 0.52 | 0.48 | 367.00 | 359.00 | 149.91 | 163.76 | 217.09 | 195.24 | 71.80 | 6.72 |
| 19 | 442.54 | 457.20 | 390.74 | 399.55 | 0.53 | 0.46 | 456.00 | 444.00 | 271.87 | 265.54 | 184.13 | 178.46 | 83.30 | 9.38 |
| 20 | 428.80 | 435.27 | 396.53 | 392.33 | 0.47 | 0.51 | 418.00 | 438.00 | 306.25 | 298.55 | 111.75 | 139.45 | 76.40 | 17.03 |
| 21 | 378.48 | 375.79 | 316.80 | 320.12 | 0.47 | 0.51 | 374.00 | 378.00 | 187.09 | 188.06 | 186.91 | 189.94 | 94.00 | 1.09 |
| 22 | 404.77 | 406.30 | 354.36 | 349.16 | 0.51 | 0.48 | 404.00 | 399.00 | 224.67 | 240.54 | 179.33 | 158.46 | 72.30 | 2.03 |
| 23 | 402.40 | 408.12 | 348.54 | 336.65 | 0.49 | 0.50 | 407.00 | 406.00 | 218.73 | 192.01 | 188.27 | 213.99 | 50.40 | 4.38 |
| 24 | 383.69 | 382.03 | 324.05 | 315.76 | 0.51 | 0.47 | 385.00 | 374.00 | 167.54 | 164.21 | 217.46 | 209.79 | 99.10 | 4.84 |
| 25 | 340.21 | 349.90 | 280.99 | 277.52 | 0.53 | 0.55 | 350.00 | 355.00 | 94.91 | 88.78 | 255.09 | 266.22 | 79.10 | 2.03 |
| 26 | 372.08 | 375.06 | 328.16 | 313.45 | 0.50 | 0.52 | 367.00 | 375.00 | 118.74 | 129.08 | 248.26 | 245.92 | 68.80 | 4.06 |
| 27 | 401.74 | 398.94 | 338.46 | 335.91 | 0.50 | 0.48 | 405.00 | 397.00 | 225.54 | 212.58 | 179.46 | 184.42 | 87.50 | 5.94 |
| 28 | 417.61 | 422.05 | 357.73 | 358.96 | 0.53 | 0.45 | 425.00 | 403.00 | 237.77 | 236.93 | 187.23 | 166.07 | 74.50 | 12.81 |
| 29 | 359.65 | 359.43 | 297.55 | 304.88 | 0.48 | 0.52 | 346.00 | 359.00 | 152.33 | 142.77 | 193.67 | 216.23 | 78.40 | 2.03 |
| 30 | 403.72 | 400.01 | 337.89 | 338.96 | 0.50 | 0.51 | 405.00 | 404.00 | 142.30 | 126.96 | 262.70 | 277.04 | 81.50 | 2.19 |
